# G2Vec: Distributed gene representations for identification of cancer prognostic genes

**DOI:** 10.1038/s41598-018-32180-0

**Published:** 2018-09-13

**Authors:** Jonghwan Choi, Ilhwan Oh, Sangmin Seo, Jaegyoon Ahn

**Affiliations:** Department of Computer Science & Engineering, Incheon National University, Incheon, South Korea

## Abstract

Identification of cancer prognostic genes is important in that it can lead to accurate outcome prediction and better therapeutic trials for cancer patients. Many computational approaches have been proposed to achieve this goal; however, there is room for improvement. Recent developments in deep learning techniques can aid in the identification of better prognostic genes and more accurate outcome prediction, but one of the main problems in the adoption of deep learning for this purpose is that data from cancer patients have too many dimensions, while the number of samples is relatively small. In this study, we propose a novel network-based deep learning method to identify prognostic gene signatures via distributed gene representations generated by G2Vec, which is a modified Word2Vec model originally used for natural language processing. We applied the proposed method to five cancer types including liver cancer and showed that G2Vec outperformed extant feature selection methods, especially for small number of samples. Moreover, biomarkers identified by G2Vec was useful to find significant prognostic gene modules associated with hepatocellular carcinoma.

## Introduction

Accurate identification of cancer prognostic genes is important in that it can lead to improvement in the accuracy of outcome prediction and better therapeutic trials for cancer patients. Many computational approaches have applied various statistical or machine learning methods to gene expression profiles to achieve this goal. For instance, Emura *et al*. proposed a copula-based application of Cox regression for selecting prognostic markers from gene expression data^1^, and Sun *et al*. exploited support vector machine, which is a renowned machine learning model, to predict cancer prognosis^2^. These approaches successfully identify individual genes that can be used for cancer prognosis, but more recent studies proposed to exploit biological network data to identify group of interacting genes that can increase the accuracy in the prediction of cancer prognosis. For example, Langfelder and Horvath explored a gene module in a weighted correlation network and selected prognostic genes with a trait-based gene significance measure^3^. Wu and Stein derived prognostic genes using the Markov Cluster Algorithm and supervised principal component analysis^4^. Google’s PageRank algorithm, which ranks websites by counting the number and quality of links to a page, was utilized to evaluate the importance of a gene in prognosis using the gene network^5^. Development of these new methods continues to improve the accuracy of outcome prediction in several cancer types. Moreover, the resulting gene module or sub-networks of genes enables better understanding of molecular mechanisms of tumor progression. However, we paid attention to the possibility that deep learning models permit more accurate prediction of cancer outcome and identification of gene networks with richer information

The recent development of deep learning has been remarkable, and it has outperformed traditional machine learning methods, especially in image and natural language processing^6–8^. A variety of deep learning models have already been employed in many bioinformatics domains, such as protein structure prediction, biomedical imaging, and biomedical signal processing^6^. Esteva *et al*. trained a Convolutional Neural Network (CNN) model using about 130,000 skin images and was able to diagnose skin cancer as accurately as a dermatologist^7^. A Recurrent Neural Networks (RNN) is renowned for analyzing enormous medical documents, and it was utilized to precisely extract medical information from unstructured text of electronic health record notes^8^. In view of the previous results, the application of the deep learning models is also expected to demonstrate excellent performance in analyzing omics data including gene expression data for identification of better prognostic genes and more accurate outcome prediction.

One of the main problems regarding the adoption of deep learning for this purpose is that, different from image or document data, cancer patient data have too many dimensions for a relatively small number of samples. Exploiting additional network data can be an efficient solution to this problem, because they can explain dependencies between genes, which can result in a similar effect to reduction of a dimension. However, it is impossible to use biological networks to learn cancer patient data using the existing DNN (Deep Neural Networks), CNN and RNN based methods without great modification.

To address this problem, we took inspiration from Word2Vec^9^ and Node2Vec^10^. Word2Vec was developed to measure syntactic and semantic word similarities among words^9^. There are two models, the continuous bag-of-words (CBOW) and the skip-gram^11^. Both provide a word vector representation that is trained from a large dataset for vocabulary and context, so the similarity between words can be easily computed by using vector algebraic operations. Node2Vec, which is a variation of Word2Vec, regards a random path generated by a random walk as a sentence or set of words. Hence, Node2Vec can capture feature representations of nodes in networks in the similar way as Word2Vec models generate vector representation of words^10^. Node2Vec has been utilized to predict protein-protein interactions^10^ or to identify disease-associated single nucleotide polymorphisms (SNPs) by using feature representations learned from networks^12^.

In this study, we propose a novel network-based deep learning method, called G2Vec, to identify prognostic gene signatures. G2Vec is a modified CBOW model and provides distributed gene representations by learning gene correlation networks regarding good and poor prognosis groups made from the functional interaction (FI) network^13^. These random paths generated from correlation networks are thought of as sentences in the context of the cancer prognosis. G2Vec is trained by predicting whether an input path was generated from a good or poor network and provides learned distributed gene representations for identifying prognostic markers. Then we computed gene scores using the gene vector representations and identified 100 biomarker genes with high scores.

We applied the proposed gene selection procedure to gene expression data for five cancer types: bladder urothelial carcinoma (BLCA), breast invasive cancer (BRCA), cervical and endocervical cancers (CESC), acute myeloid leukemia (LAML), and liver hepatocellular carcinoma (LIHC), from The Cancer Genome Atlas (TCGA). For all cancer types, we observed two clearly distinguished gene groups that represent good and poor prognosis groups when visualizing distributed gene representations, and biomarkers selected from those gene groups showed higher prediction accuracy than genes identified by existing feature selection methods when the random forest classifier was applied^14^. Gene-annotation enrichment analysis on the LIHC dataset resulted in gene modules for biological functions associated with liver cancer prognosis. These gene modules include novel prognostic biomarkers, INPP4B, RUVBL1, and HDAC1, with their roles in the progression of liver cancer. Consequently, we suggest G2Vec as an effective tool for studying cancer data, and this may form a foundation for application of deep learning for exploiting gene network data.

## Results

### Description of data sets

We downloaded five high-throughput sequencing datasets and the corresponding clinical information for BLAC, BRCA, CESC, LAML, and LIHC from the Broad Institute GDAC Firehose^15^. We used the RSEM genes normalized results (level 3) and normalized the expression values using log2 transformation after adding an offset of 1. For each cancer type, we set a criterion of survival times to manage a classification problem. The outcome of patients who survived longer than the criterion and had no death events was defined as good prognosis. In contrast, we defined the prognosis for patients with reported deaths within the criterion as poor prognosis. Table 1 shows a summary of gene expression datasets.

**Table 1:** Summary of gene expression datasets.

Cancer type	#Total samples	#Good	#Poor	#Total genes	Criterion for label
BLCA	163	77	86	16151	2 years
BRCA	165	98	67	16603	5 years
CESC	101	55	46	16272	3 years
LAML	104	41	63	14983	1 year
LIHC	135	77	58	15568	2 years

We also downloaded FI network data from the Reactome database^16^ accessed in April 2017. The FI network is derived from curated pathways, protein-protein interactions, gene co-expression, GO annotations, and text-mined protein interactions^13^. After filtering the predicted edges from the network, the number of interactions in the FI network is 298,799. We also downloaded two other gene networks, BioGrid^17^ and HumanNet^18^. The number of edges in BioGrid and HumanNet are 416,692 and 474,620, respectively.

### Visualization of distributed gene representations

**Figure 1:**
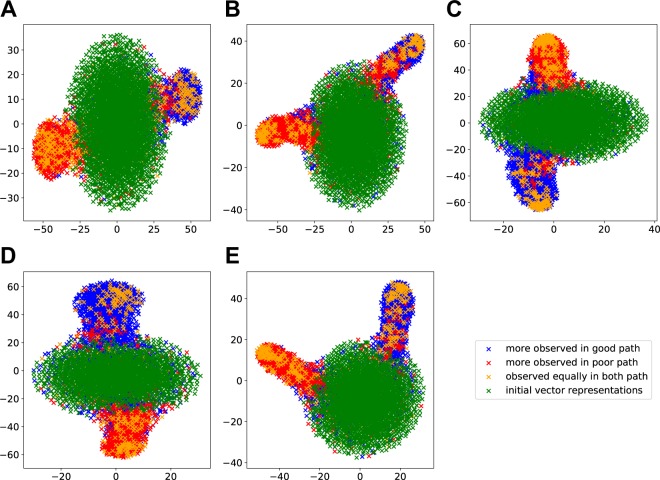
Scatter plots of distributed gene representations with t-SNE for 5 cancer types. (**A**) BLCA, (**B**) BRCA, (**C**) CESC, (**D**) LAML, and (**E**) LIHC; blue and red points represent genes more frequently occurred in the paths of good and poor prognosis groups, respectively. Yellow points indicate genes whose numbers of occurrence in two prognosis groups were equal. Green points represent genes with no occurrence in any path.

G2Vec provides numeric representations of genes, which enable quantitative analysis for genes associated with cancer prognosis. We first visualized distributed gene representation to design an effective approach for identification of prognostic gene markers by t-distributed stochastic neighbor embedding (t-SNE)^19^, which is a broadly used dimensional reduction and visualization method. Figure 1 shows all distributed gene representations computed by G2Vec. In all cancer types, we confirmed that there were three distinct gene groups, a large central vector group and two gene vector groups separated from the center. The gene vectors in the center were initial random vectors that are not updated through the training process. Gene vectors retain initial vectors because the corresponding genes were not contained in any random paths. Two trained gene groups appeared to be related to the good and poor prognosis groups, respectively. Each gene group was composed mainly of genes whose frequency in the paths generated from good or poor outcome group (Fig. 1). Genes having same label tended to be agglomerated except for yellow labeled genes, so we thought that those genes may have important information for distinguishing two prognosis groups. Moreover, these two groups tended to be far from the center of the initial vectors. Gene vectors of the two groups were tuned to discriminate between two correlation networks comprising good and poor prognosis groups through the optimization of the deep learning model in G2Vec. Hence, we assumed that the farther a gene vector is from the center, the more relevant the corresponding gene is to cancer prognosis. We applied K-means clustering algorithm with K = 3 to distributed gene representations and found the initial random vector group and two interesting gene groups that are distinguishable from the initial random vector group (Supplementary Fig. S1). We named the two distinguishable vector groups *good L-group* and *poor L-group*, respectively.

### Identification of biomarkers and outcome prediction

**Figure 2:**
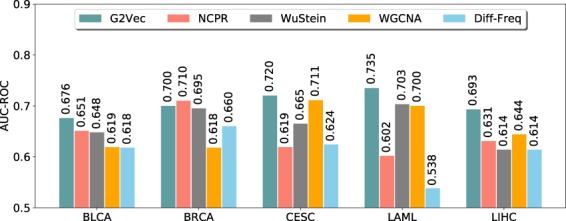
Bar plots for the accuracy for predicting patient outcomes for 5 cancer types. The x-axis represents a cancer type. The y-axis represents the prediction accuracy in terms of AUC-ROC by 10-fold cross-validation.

Using distributed gene representations, we scored genes to identify markers for predicting patient outcomes. We selected prognostic genes from each *L-group* using the *d-score*, which is the distance between vectors and the center, and the *t-score*, which measures the difference in gene expression levels between good and poor prognosis groups. We evaluated the power of biomarkers by comparing the prediction accuracy with three existing feature selection methods, NCPR^5^, WuStein^4^, and WGCNA^3^. In addition, we also selected biomarkers with high occurrence frequency in the generated paths. We named this approach Diff-Freq and selected top 50 genes from each good and poor prognosis group with the difference of occurrence frequencies in the paths, using Diff-Freq. We applied these feature selection methods to five sets of gene expression data from TCGA and predicted patient outcomes using the random forest classifier. We conducted 10-fold cross validation and computed the prediction accuracy in terms of the area under the receiver operating curve (AUC-ROC). The results of the prediction accuracy are shown in Fig. 2. We confirmed that the performance of G2Vec was higher than the others (0.009–0.049) in most cancer types, with the exception of BRCA. In BRCA, G2Vec was not the best, but it still showed good accuracy.

We also applied G2Vec to BioGrid and HumanNet in addition to the FI network. Supplementary Fig. S2 showed the AUC-ROC values using FI were slightly higher than other networks, but differences were not significant in all cancer types, which means that G2Vec works for any gene network without bias. Following experiments were conducted with FI network.

### Effective sample size for G2Vec

**Figure 3:**
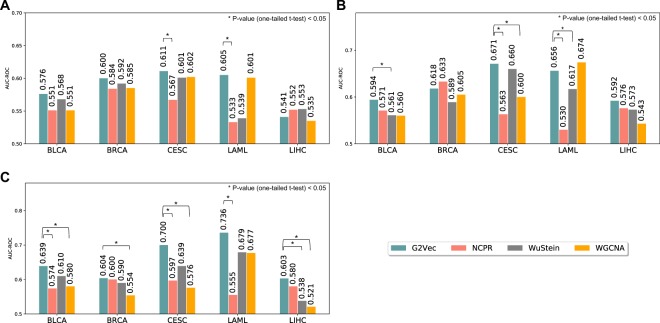
Bar plots for the comparison between gene selection methods with small training datasets. The average AUC-ROC computed from 10 datasets of each cancer type for (**A**) 20, (**B**) 40, and (**C**) 60 training samples. The significant difference of AUC-ROC between methods was computed using one-tailed t-test.

Next, we checked the effective sample size of G2Vec to show that G2Vec is effective for small number of gene expression profiles. For each cancer type, we generated 10 sets of training and test data. Training data is composed of 10, 20, or 30 samples that are randomly chosen from each prognosis group, and the remainder samples were used as test data. In the similar way as in the previous experiments, we computed prediction accuracy of G2Vec and the extant methods on generated small datasets. Figure 3 shows that G2Vec outperformed the existing methods in most cases. When using 60 samples, G2Vec showed similar accuracy to the cases when using whole samples. Taken together, we can say that G2Vec has strength in handling a small number of samples.

### Identification of prognostic modules from gene networks

**Figure 4:**
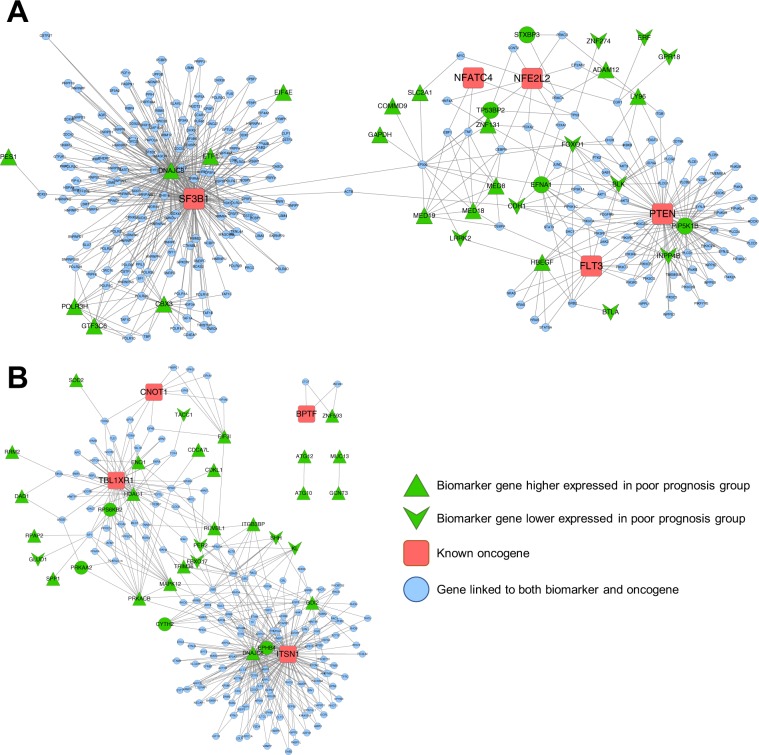
Subnetworks with biomarkers and oncogenes for LIHC. (**A**) *Good L-group* specific subnetwork of FI network; (**B**) *Poor L-group* specific subnetwork of FI network; Red, green, and blue genes stand for oncogenes, biomarkers, and the others, respectively. Triangle and V genes represent genes that are expressed higher and lower in poor prognosis group than in good prognosis group, respectively.

Distributed gene representations can be used to find significant prognostic gene modules associated with cancer prognosis. We identified prognostic modules associated with liver cancer using 100 biomarkers identified by G2Vec (Supplementary Table S1) and 9 known oncogenes associated with hepatocarcinoma that were downloaded from the IntOGen database^20^. We first created two subnetworks from the entire FI network to investigate good and poor *L-groups*. All edges having at least one of the biomarkers or oncogenes were collected, and genes linked with only one among the biomarkers and oncogenes were removed. The *good L-group* and *poor L-group* subnetworks consisted of 280 and 198 genes, respectively (Supplementary Table S2); these subnetworks are shown in Fig. 4. Next, we carried out gene-annotation enrichment analysis using DAVID 6.8 database^21^ to explore biological functions or pathways in each subnetwork and identified 104 and 60 significant Gene Ontology (GO) terms and KEGG pathways (Supplementary Table S3) from the *good L-group* and *poor L-groups*, respectively. We then conducted the log-rank test^22^ on biomarkers contained in these GO terms or pathways to identify prognostic gene modules. For the other cancer types, the lists of GO terms and KEGG pathways were provided in Supplementary Tables S4–S7.

**Figure 5:**
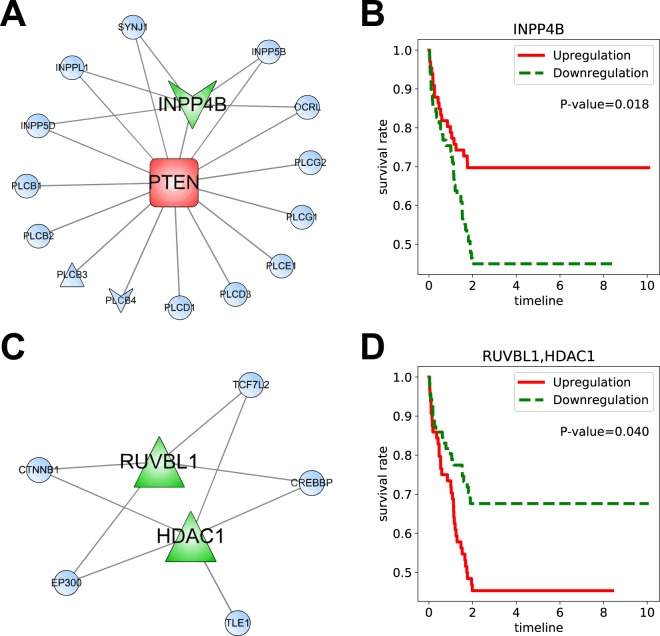
Significant prognostic gene modules. (**A**) Subnetwork of GO:0043647 from *good L-group* specific subnetwork; (**B**) Kaplan-Meier graph of GO:0043647; (**C**) Subnetwork of GO:1904837 from *poor L-group* specific subnetwork; (**D**) Kaplan-Meier graph of GO:1904837; In the Kaplan-Meier graph, High expression indicates the patient group whose INPP4B (or the mean of RUVBL1 and HDAC1) expression value is higher than the average of expression of the gene on the whole patients. Low expression represents the patient group whose gene expression value is lower than the average. P-values denoted in Kaplan-Meier plots are computed with log-rank test.

Using the aforementioned procedure, we found two significant prognostic modules, GO:0043647~inositol phosphate metabolic process and GO:1904837~beta-catenin-TCF complex assembly from the *good L-group* and *poor L-group* subnetworks, respectively. These modules and their results in terms of survival analysis are shown in Fig. 5. The Kaplan-Meier plots in Fig. 5 show that the high expressed inositol phosphate module and the low expressed beta-catenin-TCF complex represent good prognosis in patients with liver cancer. Inositol phosphates are known to play crucial roles in various cellular functions, such as cell growth, apoptosis, and cell differentiation. In particular, the INPP4B gene, identified as a biomarker of LIHC by G2Vec, has been studied as a tumor suppressor in various cancer types, such as breast, ovary, and prostate cancers^23^. The overexpression of INPP4B suppresses the PI3K/AKT signaling pathway and results in reduced tumor growth, which appears to be associated with PTEN^24^. This result may support our finding that low expression of inositol phosphates with INPP4B is associated with poor prognosis of LIHC. In addition, the beta-catenin-TCF complex has been broadly researched as a therapeutic target for colorectal and gastrointestinal cancer^25,26^. In the liver, the beta-catenin-TCF complex is known to suppress HNF4-alpha, which plays an essential role in development and organogenesis^27^, and HNF4-alpha is suggested to be an inhibitor of hepatocellular carcinoma^28^. These results may suggest that overexpression of the beta-catenin-TCF complex is associated with poor prognosis of liver cancer. Thus, we expect that the inositol phosphate and beta-catenin-TCF complexes would play key roles in cancer prognosis.

## Discussion

In this paper, we propose a network-based deep learning model called G2Vec to identify prognostic biomarkers and gene modules. G2Vec showed significantly higher prediction accuracy for patient outcomes than existing gene selection methods. In addition, we found significant prognostic modules associated with hepatocellular carcinoma using biomarkers identified by G2Vec.

While general deep learning models require a large number of training samples, we showed that G2Vec is effective for small number of training samples (Fig. 3). This is because G2Vec generates many random paths from a correlation network. In our experiments, G2Vec generated about 43,000 paths (Supplementary Fig. S3), which is the number of inputs enough large to train a deep learning model, from gene expression data with sample size of about 130.

G2Vec has a couple of issues in training process. First, optimal parameters of G2Vec were obtained by heuristic approach. G2Vec has three critical parameters: length of random paths, size of embedded representations, and learning rate. Optimal parameters are ones that results in highest prediction accuracy of modified CBOW. The optimal maximum length of a random path, the size of projection layer, and learning rate were set to 80, 128, and 0.005, respectively (Supplementary Fig. S4). Although these values showed the best accuracy in our experiment, optimal parameters can be changed on different datasets. Second, we exploited early stopping to avoid overfitting^29^. In all cancer types, the modified CBOW was trained within 30 epochs, which may be too small to learn network structure completely. However, we confirmed that G2Vec showed good performance using the small epochs in Figs 2 and 3, which means that the number of epoch is sufficient. In addition, the small number guarantees fast processing time, which is another merit of G2Vec.

G2Vec is a deep learning-based method that learns the network structure, and we used it to analyze gene expression data. However, G2Vec can be extended for another omics data such as SNPs, copy number variation, or DNA methylation data, because G2Vec can be applied to any kinds of data that can be used to make correlation networks. In the future, we plan to apply G2Vec to multiple omics datasets and develop integrative analysis method for cancer research.

## Methods

### Overview of gene selection and validation process

**Figure 6:**
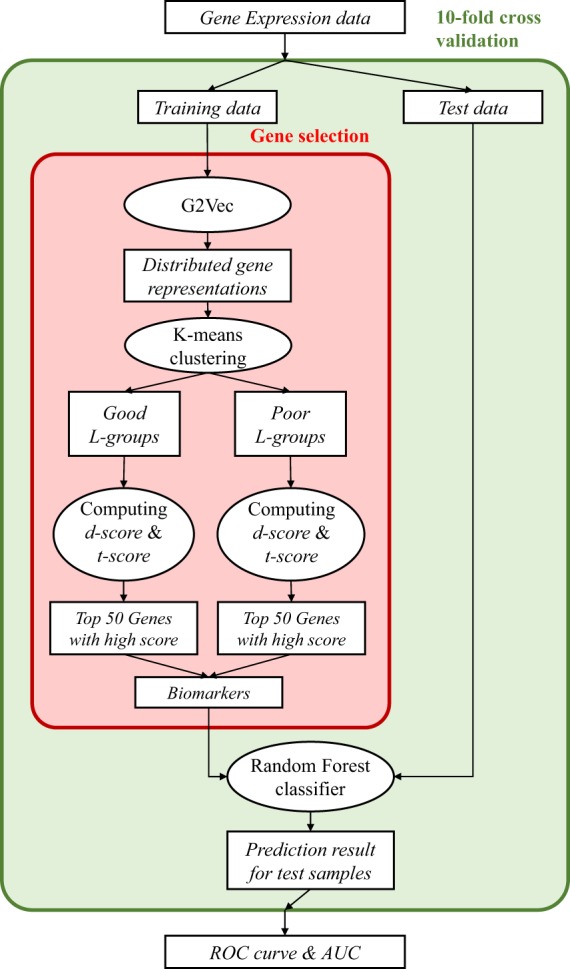
Overview of biomarker selection and prognosis prediction. Processes within green box are repeated for each fold. Processes within red box show our proposed gene selection procedure.

The proposed gene selection method consists of three steps, generating distributed gene representations, finding *L-groups*, and computing gene scores. Then prognosis is predicted through 10-fold cross validation using the random forest classifier. Figure 6 shows an overview of biomarker selection and prognosis prediction. G2vec was implemented in Python3 with tensorflow and scikit-learn modules. The source code is freely available on GitHub at https://github.com/mathcom/G2Vec.

### Distributed gene representations

G2Vec is a modified CBOW model to compute distributed representations of genes. From each gene correlation network from the good and poor prognosis groups, random paths are generated using a random walk algorithm. These random paths are thought of as sentences from the context of good or poor outcome groups and used for learning distributed gene representations that distinguish good and poor groups by G2Vec.

**Figure 7:**
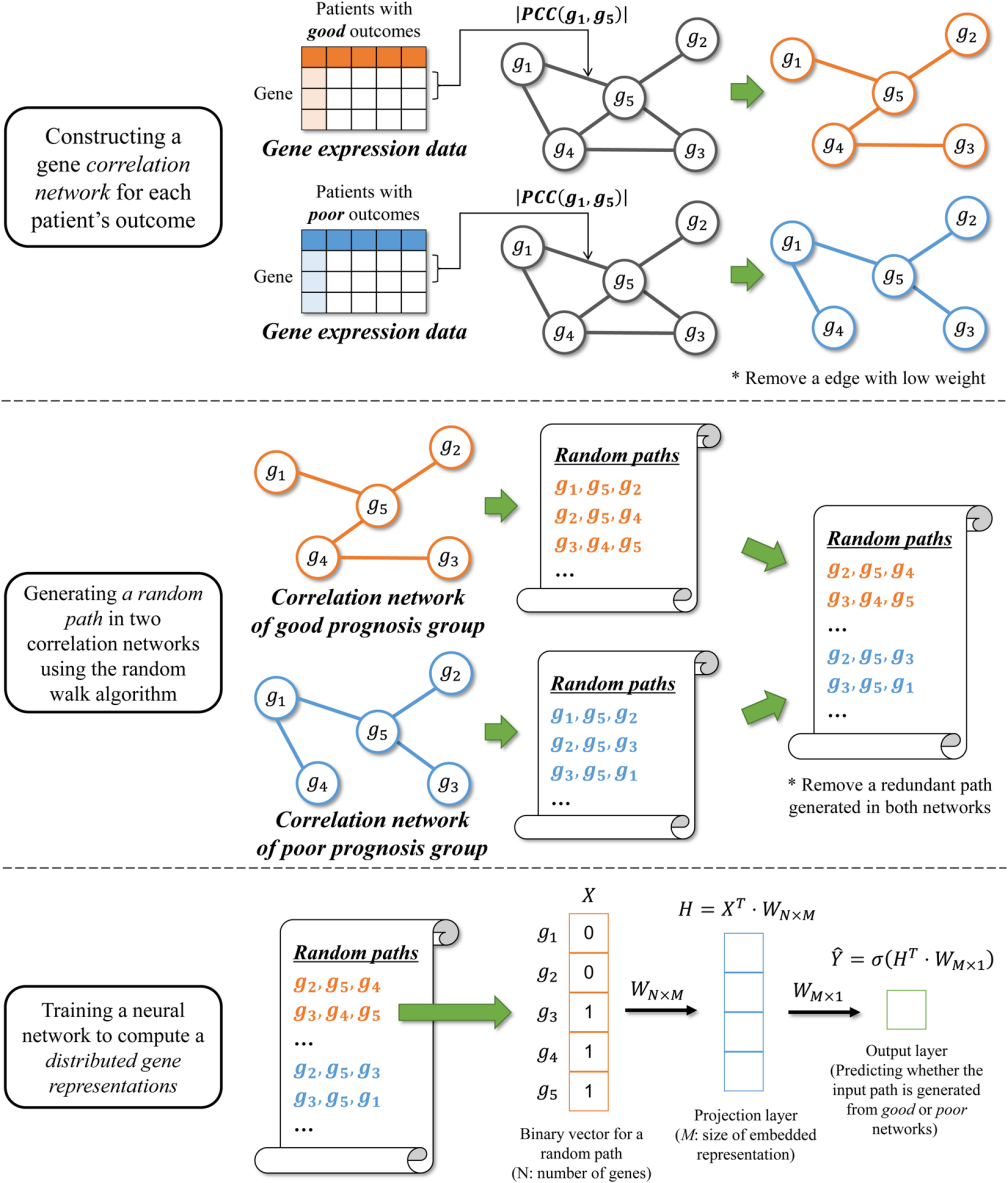
Overview of G2Vec. G2Vec has three steps: constructing correlation networks, generating random path, and training a modified CBOW model. The trained weights *W*_*N*×*M*_ between the input and projection layer are used as gene vector representations.

G2Vec has three steps, constructing correlation networks, generating random paths, and training a neural network. An overview of G2Vec is shown in Fig. 7.

First, we created two correlation networks associated with good and poor outcomes from the FI network and gene expression data. For each outcome group, we computed an absolute value of Pearson’s correlation coefficient (PCC) between expression levels of two genes linked on the FI network, and the values were used as a weight of the interaction. Some interactions with weight <0.5 were removed to make the difference between the two correlation networks clearer.

Next, we applied the random walk algorithm to each weighted correlation network, with three constraints. First, we never revisited a node that was passed. Second, a next destination was determined proportionally to the weight of edges. Lastly, we stopped when reaching a dead end or when the length of a path is equal to a pre-defined maximum length of the path. In this study, the maximum length of a path was set to 80. We departed from all genes 10 times, and paths were collected to learn a neural network. We gathered numerous random paths from correlation networks of good and poor prognosis groups and removed a redundant path generated in both networks. Among the filtered paths, 80% and 20% are used as training and validation datasets, respectively.

Last, we trained a modified CBOW neural network model with one hidden layer, called a projection layer. A path was represented as a binary vector whose size was equal to the number of genes. The value of a binary vector indicates whether a gene is contained in a random path or not. A neural network predicts whether an input path is generated from good or poor prognosis groups. The neural network is trained using the cross entropy objective function and Adam optimization algorithm^30^. Early stopping with validation set was used to avoid overfitting. We heuristically set the optimal parameters of a neural network, such as number of projection neurons and learning rate (Supplementary Fig. S4). In this study, we used 128 projection neurons and learning rate of 0.005. The trained weights between the input and projection layer (*W*_*N*×*M*_ in Fig. 7) were used as gene vector representations.

### L-group and gene scores

Distributed gene representations generated by G2Vec were used to group genes and to compute gene scores for identification of prognostic biomarkers. Gene vectors formed two gene groups associated with either good or poor outcome groups (Fig. 1). These groups named *L-groups* could be detected with K-means clustering algorithm (Supplementary Fig. S1). We then selected prognostic biomarkers from each *L-group* with gene scores. A gene score was defined by the means of *d-scores* and *t-scores*. A *d-score* is the Euclidean distance between a gene vector and the center of initial gene vectors (zero vector). A *t-score* is the absolute value of t-statistics measuring the difference of gene expression levels between good and poor outcomes. Both scores were normalized from 0 to 1 by min-max transformation. We selected 50 genes with high gene scores from each *L-group*, resulting in 100 biomarkers. For each fold, 100 biomarkers were identified using training data and validated with test data using the random forest classifier.

## Electronic supplementary material

Supplementary Figures

Supplementary Tables
