# Supplementary material for: G2Vec: Distributed gene representations for identification of cancer prognostic genes
Source: Sci Rep. 2018 Sep 13;8:13729. doi: 10.1038/s41598-018-32180-0 (PMC6137174; doi:10.1038/s41598-018-32180-0)
Supplement: Supplementary file 1 — Supplementary Figures [file 41598_2018_32180_MOESM1_ESM.pdf]

G2Vec: Distributed gene representations for identification of cancer prognostic genes

Jonghwan Choi, Ilhwan Oh, Sangmin Seo, Jaegyoon Ahn*

- Supplementary Figures -

Figure S1. Results of K-Means clustering applied to distributed gene representations.

Figure S2. Receiver operating characteristic curves of networks per cancer types.

Figure S3. Distribution of path lengths per prognosis groups.

Figure S4. The line plots per parameter for selecting optimal values

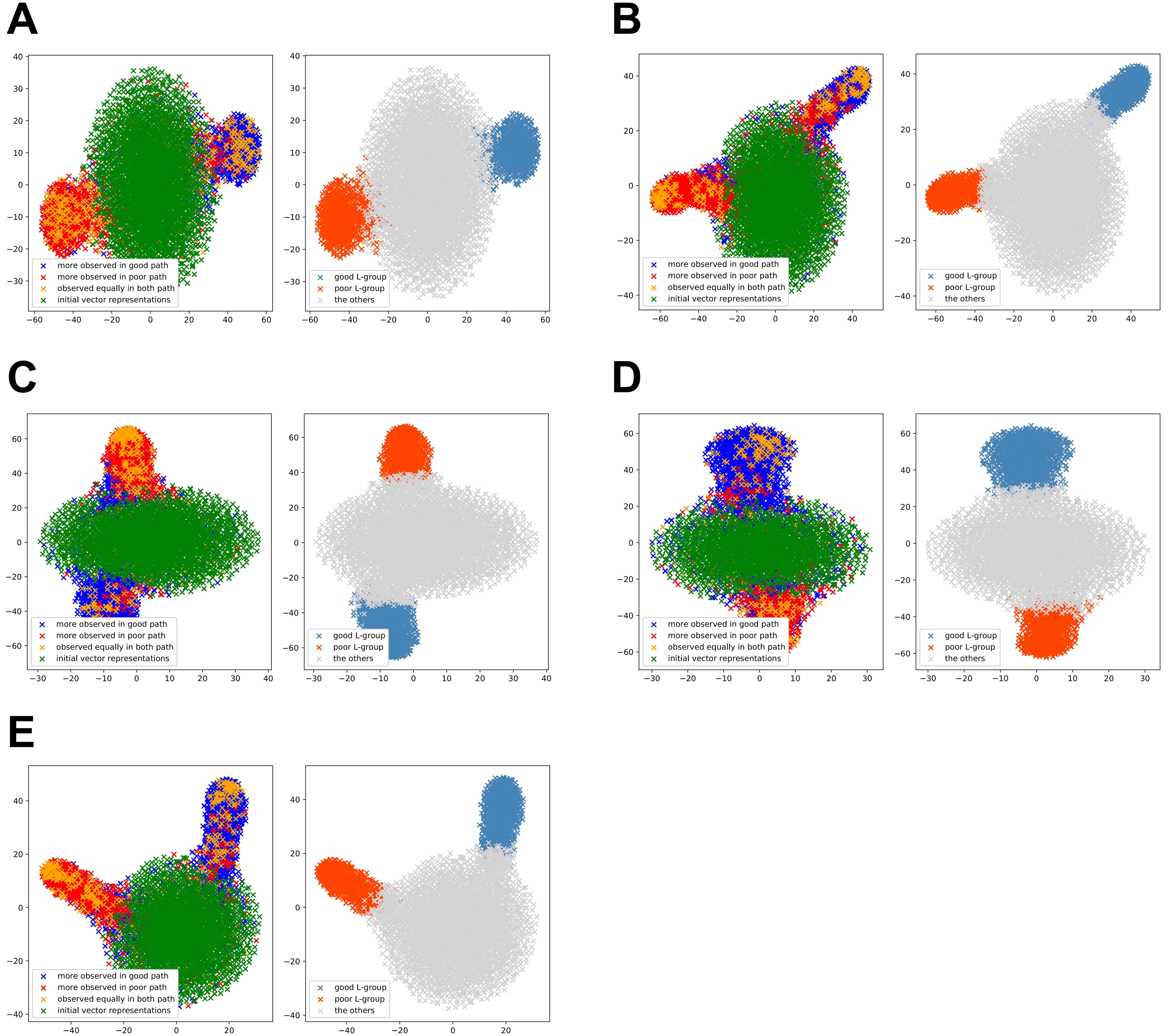

Figure S1. Results of K-Means clustering applied to distributed gene representations. (A) BLCA, (B) BRCA, (C) CESC, (D) LAML, and (E) LIHC; For each cancer type, the left represents the relationship between distributed gene representations and cancer outcome groups, and the right shows L-groups detected by K-Means clustering; In each left plot, a gene is labeled as “more observed in good path” if the number of occurrence in the random paths of good prognosis group is larger than one in poor prognosis group. In a similarly way, a label “more observed in poor path” is assigned. A label “observed equally in both path” represents a gene whose numbers of occurrence in good and poor prognosis groups are equal. A gene with no occurrence in any path is labeled as “initial vector representations”.

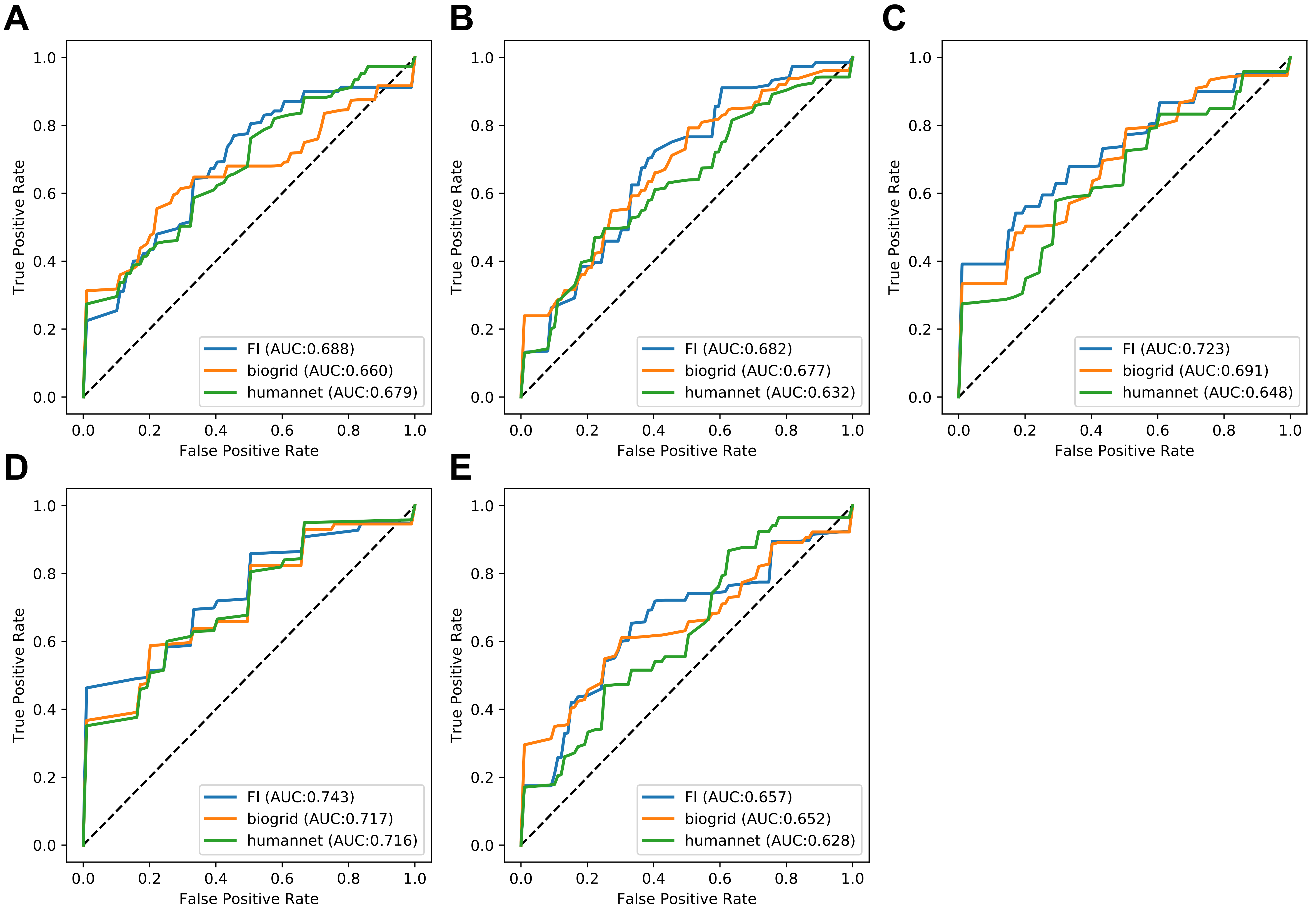

Figure S2. Receiver operating characteristic curves of networks per cancer types. (A) BLCA, (B) BRCA, (C) CESC, (D) LAML, and (E) LIHC

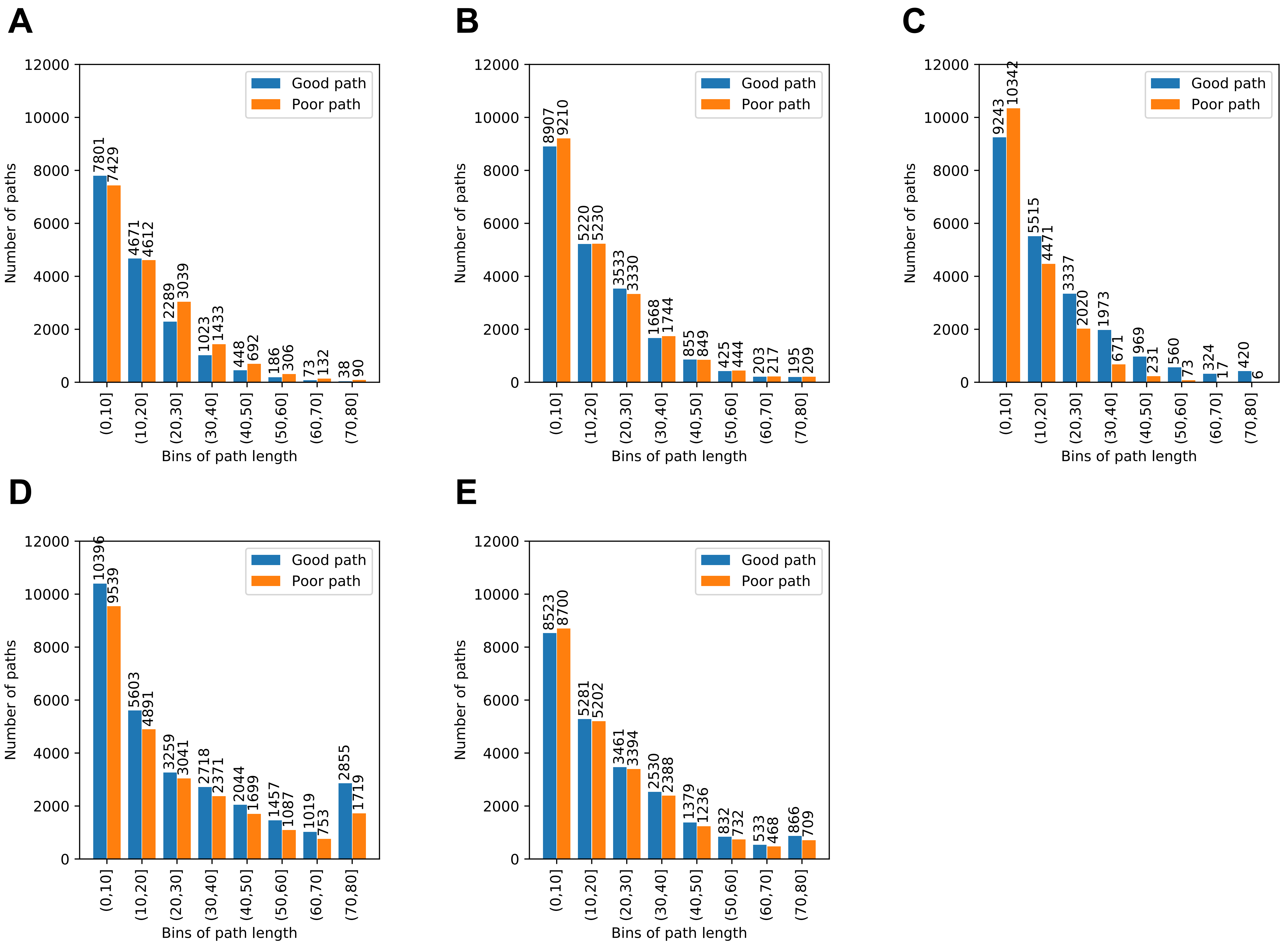

Figure S3. Distribution of path lengths per prognosis groups. (A) BLCA, (B) BRCA, (C) CESC, (D) LAML, and (E) LIHC; x-axis and y-axis represent bins of path length and the number of paths in bins, respectively.

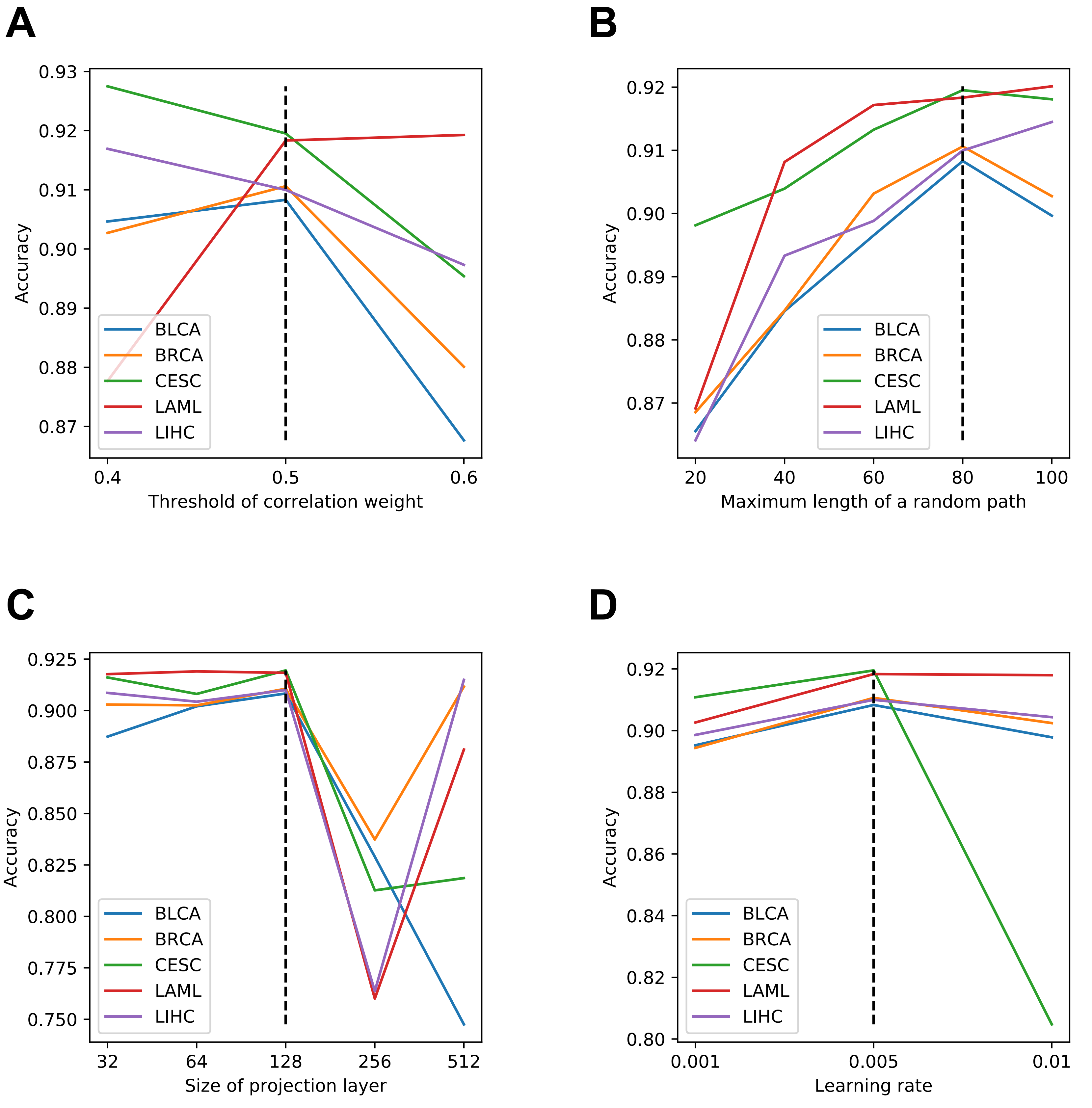

Figure S4. The line plots per parameter for selecting optimal values. (A) Threshold of correlation weight, (B) Maximum length of a random path, (C) Size of projection layer, and (D) Learning rate; The y-axis represents the prediction accuracy of G2Vec predicting whether a random path was generated from correlation networks of good prognosis group or poor prognosis group. In each plot, other parameters were fixed the identified optimal parameters. The dash line indicates the selected optimal value
